# Effects of Stockpiling on Topsoil Biogeochemistry for Semiarid Mine Reclamation

**DOI:** 10.1007/s42461-024-01164-2

**Published:** 2025-01-08

**Authors:** Jessica Ledesma, Julia W. Neilson, Raina M. Maier, Alicja Babst-Kostecka, Craig Rasmussen

**Affiliations:** 1 The Department of Environmental Science, The University of Arizona, Tucson, AZ USA; 2 Geosystems Analysis, Inc., Tucson, AZ USA

**Keywords:** Topsoil stockpile, Copper mining, Mine reclamation, Soil biogeochemistry, Redox processes, Semiarid climate

## Abstract

Stockpiling and storage of topsoil for use in reclamation and revegetation are common practices for many mining operations. However, stockpiling can lead to significant changes in topsoil physical and biogeochemical properties that may be detrimental to reclamation. The objective of this research was to assess the effect of long-term stockpiling on soil biogeochemical properties in a semiarid region. We hypothesized that soil properties would change systematically with depth reflecting a shift to anaerobic conditions and resulting in a general decrease in soil health. To address this hypothesis, boreholes > 20-m deep were drilled into a 14-year-old topsoil stockpile at a copper mine in Arizona and samples collected every ~ 75 cm. Samples were analyzed for soil DNA biomass, texture, general agronomic properties, mineral composition, oxalate and dithionite extraction of active mineral phases, and total elemental composition. Depth profiles revealed non-systematic changes in biogeochemical variables with depth, including variation in soil DNA biomass, organic matter (OM), extractable nitrate (NO_3_-N) and ammonium (NH_4_-N) nitrogen, plant-available manganese (Mn) and iron (Fe), and oxalate-extractable Mn and Fe. Differences in biogeochemical properties were associated with zones of variable redox state mediated by OM content and layer depth. Anaerobic zones were observed at depths greater than 4 m where OM > 1%, and aerobic zones were observed at depths up to 15 m where OM < 1%. This study demonstrates the importance of stockpile composition on biogeochemical processes during storage and contributes to improved understanding of topsoil management as a resource for reclamation of degraded mine lands in semiarid environments.

**Supplementary Information:**

The online version contains supplementary material available at https://doi.org/10.1007/s42461-024-01164-2.

## Introduction

Stockpiling and storage of topsoil materials for mine-land restoration are common practices in mining operations globally [1]. The use of stockpiled materials varies regionally and with type of mining operation and includes using the material to cap, stabilize, and revegetate tailings [2]. Pronounced changes in topsoil properties such as soil structure [3], biogeochemical composition and nutrient speciation [4–6], and microbial community composition and abundance [7–12] during stockpile storage often lead to a reduction of soil health and can limit the effectiveness of those materials for reclamation [13]. Past work has examined stockpiling and storage effects on topsoil biogeochemical properties in numerous environments including studies from wet, temperate environments of British Columbia [4]; warm, humid systems of Appalachia [14]; cool, semiarid systems of Wyoming [15] and the Northern Great Plains [9]; and warm, semiarid environments of Western Australia [1]. However, there is currently limited to no data on stockpiling effects on topsoil properties for the arid and semiarid regions of the Southwestern USA where mine operations are predominantly typified by large open pit mines extracting copper (Cu), associated metals, and rare earth elements [16]. This study is aimed at addressing this important knowledge gap by quantifying biogeochemical changes in a 30-m-deep 14-year-old topsoil stockpile produced during Cu mine operations in Arizona, USA.

The use of Cu for electricity and other technologies has increased production on a global scale, with ~ 2.1 billion tons of Cu in current reservoirs and 3.5 billion tons in undiscovered reserves [17]. Additionally, with the rise in green technologies such as electric vehicles, Cu usage is projected to increase by 17% in the USA alone by the year 2070 [18]. However, Cu mining can be destructive and detrimental to the environment. Copper mining produces the largest percentage of mining and processing wastes in the United States with an estimate of 1000 + acres of land used for waste storage per mine [19]. As of 2022, there were 16 active Cu mines and roughly 400 total active mines in Arizona, resulting in over ~ 16,000 acres of mining waste [16]. Legacy mine sites that utilized historical extraction methods have left behind mine tailing piles with elevated levels of heavy metals such as lead and arsenic that pose a risk to human health [20–23]. Modern mine tailings no longer create heavy metal hazards, but tailings dust emissions and recovery of the degraded lands are still a concern [24]. It is imperative to create new and innovative environmental solutions to support mine land reclamation and protect human health while still meeting demands for Cu usage.

Stockpiling of topsoil for subsequent use as material to cap mine waste is a common method associated with mine-land reclamation. Stockpiling involves scraping the topsoil from the area prior to mine development, and storing it in large, constructed stockpiles for months or years before use for reclamation [25]. Topsoil is considered the most viable section of the soil for capping materials because of its higher nutrient and organic matter contents [11, 26–28]. Ghose [29] described a “shelf-life” of soil stockpiles in which the material is biologically productive and useful for revegetation for only certain storage durations. This concept may be helpful to indicate the threshold of nutrient availability and microbial activity after which the stockpiled topsoil can no longer be reused to establish vegetation without the addition of amendments. However, the ideal “shelf-life” needed to maintain proper nutrient availability to establish vegetation may vary greatly with stockpile depth, age, and climate regime.

Given the importance of stockpiled topsoil for plant establishment in mine reclamation efforts, the particularly challenging conditions of arid and semiarid environments, and the vast expanses of Cu mines requiring reclamation in the Southwest USA, the successful implementation of effective revegetation strategies has become an imperative and demanding task. However, little to no information exists on stockpile conditions typical of Cu mine operations in the Southwest, e.g., stockpiles deeper than 3 m that remain in place for decades. This leaves a critical knowledge gap in the efficacy of topsoil stockpiling to rehabilitate Cu mine waste in arid and semiarid environments. The objective of this research was to quantify topsoil physical, microbial, and biogeochemical properties after 14 years of storage in a large 30-m-deep stockpile located in an Arizona Cu mine to understand the effect of depth and storage conditions on stockpile topsoil change in a semiarid region. To our knowledge, this is the first study to examine decadal scale stockpiling effects on topsoil biogeochemical properties in the semiarid environment of Arizona.

## Materials and Methods

### Site Description

Samples were collected from a Cu mine near Globe, Arizona, USA (reporting of the exact location and operator is limited by company policy). The area has a semiarid climate, with an average rainfall of approximately 50 cm annually, a mean annual temperature of 16.5 °C, and an aridity index, defined as the ratio of precipitation to potential evapotranspiration, of 0.22 [30, 31]. Vegetation in the area includes a mix of scrub and woodland, with overstory species consisting of mesquite, one-seed juniper, pinyon pine, and oaks, and an understory mix of grasses, yucca, manzanita, mountain mahogany, prickly pear cacti, and agaves [32]. Soils in the area are mapped as Aridic Paleustalfs, Aridic Lithic Ustorthents, Aridic Haplusteps, and Aridic Haplustalfs formed from a mix of sedimentary and igneous rocks [33].

### Sampling Methods

**Fig. 1:**
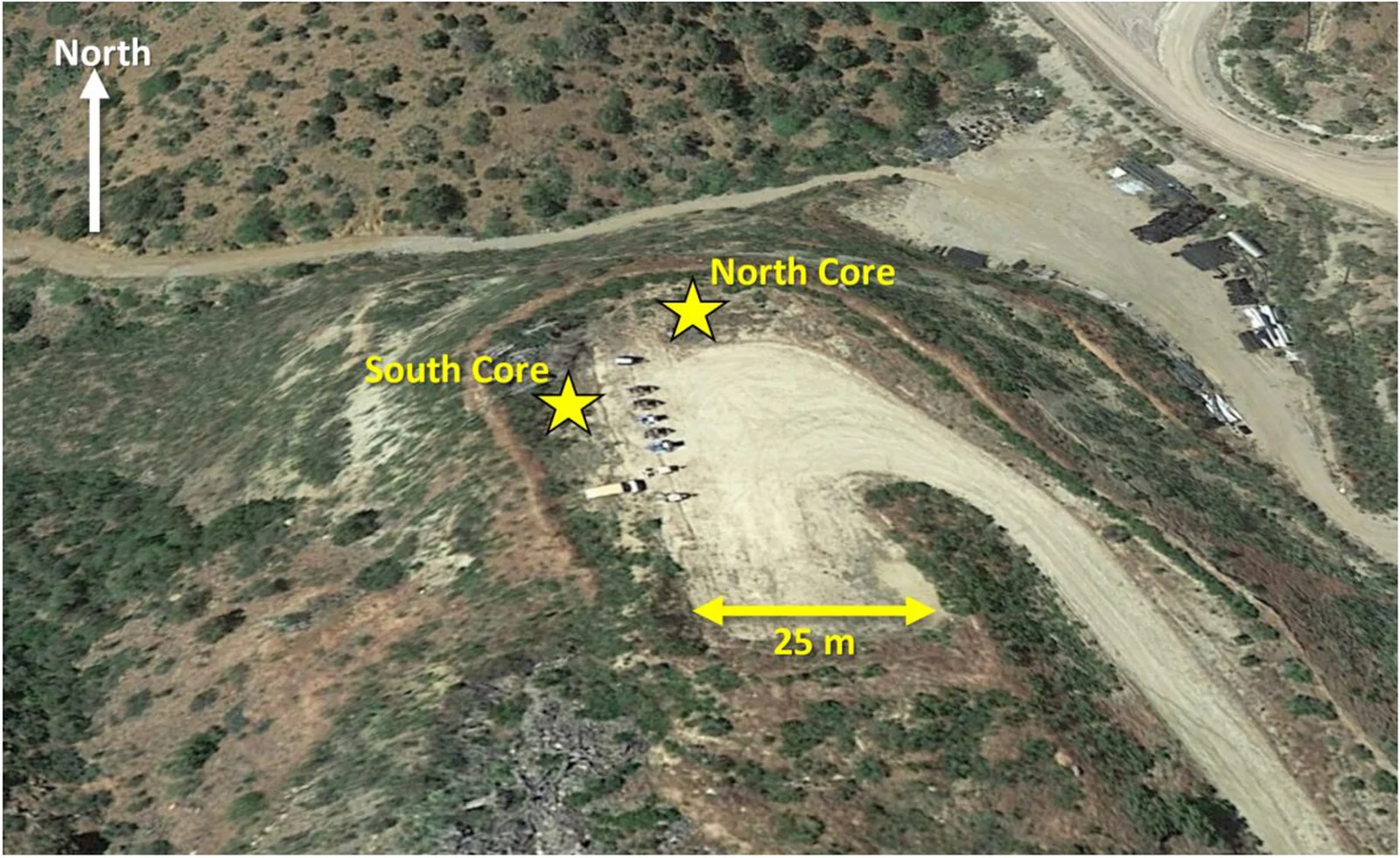
Aerial photograph of topsoil stockpile sample site near Globe, Arizona, USA. The north and south borehole locations are noted and labeled, and a scale bar of 25 m is provided for reference. Image from Google Earth with imagery date of 05/03/2019

Samples were collected in May of 2021 from a 14-year-old topsoil stockpile that was constructed in 2007. A sonic drill rig was used to core two boreholes 10 cm in diameter. The two boreholes were approximately 25 m apart. On the north side of the topsoil stockpile, the borehole was drilled to a depth of 30 m and labeled as the north core (NC) (Fig. 1). The south borehole was drilled to a depth of 20 m and labeled as the south core (SC). The coring rig removed 3-m sections and expelled the core material into 75-cm sub-cores. After the sub-cores were expelled from the drill into a plastic sleeve, the plastic was carefully cut open with a soil knife that was sterilized for 60 s with Clorox bleach. The last sub-core of the north borehole reached into the native soil below the stockpile, and the last three sub-cores from the south borehole were the native bedrock. These samples were excluded from the biogeochemical analyses and results (see Fig. S1 for the flowchart outlining sampling methods).

Microbial samples were collected in triplicate from each sub-core. These samples were collected 10 cm from the top of the sub-core, in the center of the sub-core, and 10 cm from the bottom of the sub-core using sterilized scoopulas. The samples for biological analysis were placed in 50-mL sterile plastic, conical Falcon tubes and stored in a cooler with ice packs until transported to the laboratory for storage at − 80 °C after sample processing. In total, 114 microbial soil samples were collected from the north core, and 78 soil microbial samples were taken from the south core.

Soil samples for chemical and mineralogical analyses were randomly collected from the entire length of the sub-core. These samples were sieved through a 4.75-mm sieve to separate large rocks from soil material and the rock weight recorded in the field. Sieved samples were homogenized and collected into two Whirlpack packs totaling about 1700 g of soil per sample. In total, 38 soil samples were collected from the north core, and 26 soil samples were collected from the south core. Gravimetric moisture content was determined prior to soil storage by weighing approximately ~ 15 g of soil prior to and after oven drying for 24 h at 105 °C.

### Sample Preparation

Soil microbial samples were prepped within 24 h of sample collection. Soil was sieved through a 2-mm sieve and weighed to determine gravimetric moisture content. The remaining soil in the 50-mL tubes was transferred using sterile technique into 15-mL sterile conical tubes and stored in a − 80 °C freezer until soil DNA extractions were performed.

Soil biogeochemical and mineralogical samples were air dried for 7 days in paper bags under a hood, crushed with a mortar and pestle to break up soil clods, and sieved to pass through a 2-mm sieve. Soil samples were then stored in a 4 °C cold room until prepped and shipped to commercial labs for analysis.

### Soil DNA Biomass

Soil DNA was extracted from 0.5 g of soil sample using the MP Bio FastDNA Spin Kit with modifications to the standard manufacturer’s protocol (MP Biomedicals, Solon, OH, USA) as defined previously by Kushwaha et al. [34]. Cells were lysed using a vortex at high speed for 15 min. DNA extraction samples were quantified using a Qubit® 2.0 Fluorometer (Thermo Fisher Scientific, Waltham, MA) and Qubit® dsDNA High Sensitivity Assay Kit (Life Technologies, NY, USA). A reagent blank was run with all DNA extractions, and only DNA extractions performed with a reagent blank that registered below the fluorometer DNA detection limit of 0.015 ng/μL were used in further analysis [32]. Sample DNA biomass was calculated from DNA extract concentrations and sample soil was adjusted for the soil moisture content. Biomass was expressed as nanograms of DNA per gram of dry soil (ng/g) (Table S1).

### Soil Nutrient Analysis

Samples were analyzed by Brookside Laboratories, Inc. (New Bremen, Ohio) following standard methods. Soil test packages included pH 1:1 in water; organic matter by loss on ignition; Olsen phosphorus; ammonium acetate exchangeable cations at pH 8.1; exchange capacity by sums of cations; % base saturation; DTPA extractable Fe, Mn, Cu, Zn; KCl extractable nitrogen (NO_3_-N + NH_4_-N); water-extractable nitrogen; water-extractable organic carbon; soil texture by hydrometer analysis; total organic carbon (after acid treatment); total nitrogen; and carbon/nitrogen ratio (Table S1).

### Aggregate Stability

Soil aggregate stability was analyzed using an Eijkelkamp soil and water wet sieving apparatus (Royal Eijkelkamp, Wilmington, NC). Two grams of < 2 mm air-dried soil was weighed out into aggregate sieves of 250 μm and placed in the sieve holder on the apparatus. Seventy milliliters of DI water was added to each sieve to pre-soak the soil. The soil apparatus ran for 3 min to separate the aggregate fraction < 250 μm. Seventy milliliters of sodium hexametaphosphate was added to disperse the remaining soil, and the apparatus ran for eight additional minutes. Separated aggregate fractions were rinsed into a weigh dish and placed in an oven at 110 °C for 48 h. Once the samples were fully dried, aggregate fraction samples were weighed and the percentage mass in each fraction was calculated (Table S2).

### X-ray Diffraction Analyses

The sieved soils were analyzed using quantitative X-ray diffraction to determine the percent composition of primary and secondary phases using the internal standard technique [35, 36]. Corundum was used as the internal standard in all measurements. Prior to mineralogical analyses, samples were pretreated to remove organic matter using NaOCl adjusted to pH 9.5 [37]. Twenty grams of the < 2 mm soil was mixed with 100 mL of NaOCl in a beaker and heated in a hot water bath at 80 °C for about 1 h until bubbling ceased. After the solution was cooled, samples were centrifuged, and the supernatant was discarded. The samples were then briefly shaken by hand with deionized water and centrifuged to ensure NaOCl salts were rinsed from the sample. Samples were left to dry under a hood.

The pre-treated samples were ground to less than 180 μm using a McCrone soil micronizing mill (The McCrone Group, Inc., Westmont, Illinois) and a wet grinding method. Four milliliters of ethanol was added to the micronizing container with exactly 0.5 g of oven-dried corundum used as a reference mineral and 2.0 g of pretreated soil sample. The sample and reference corundum were then ground for 5 min. Seven milliliters of ethanol was added to the container and ground for an additional 15 s. This was repeated three times and each time the liquid sample was poured into a beaker between each run. This ensured that the soil was completely removed from the grinding container. Samples were left under a flow hood to evaporate off the ethanol and then run through a 180-μm sieve. Samples were prepped as random powder mounts and measured from 5 to 65° 2-theta using a PANalytical X’Pert PRO-MPD X-ray diffraction system (Malvern Panalytical, Almelo, AA, The Netherlands) fitted with a graphite monochromator and sealed Xenon detector and producing Cu–Kα radiation at an accelerating potential of 45 kV and current of 40 mA. Mineral phases were identified using the Panalytical Highscore Plus software package, and quantitative analyses were performed using RockJock [38] (Table S3).

### Selective Dissolution and Total Elemental Analysis

Two selective dissolution soil extractions were performed to estimate the partitioning of Fe, Al, Si, and Mn to various crystalline and short-range-order mineral phases. The selective dissolutions included a citrate-dithionite extraction, whose extracts oxidized forms of Fe- and Mn-oxyhydroxides and associated Al and Si substituted into those phases, and acid ammonium oxalate, which extracts short-range-order phases of Fe-oxyhydroxides and aluminosilicates [39]. Before each extraction, any soil magnetite was removed by passing a magnetic stir bar through the soil sample. For the citrate dithionite extraction, 0.75 g of each soil sample was placed in a 50-mL sterile plastic Falcon tube with 0.4 g of sodium dithionite and 25 mL of 0.57 M sodium citrate solution. Each sample was initially shaken by hand and then placed on a reciprocal shaker for 16 h. After shaking, the samples were left upright to settle for 24 h and centrifuged at 4000 rpm for 15 min, and supernatant was separated into new sterile 50-mL Falcon tubes. The concentrations of Fe, Al, Si, and Mn were determined using ICP-OES.

Ammonium oxalate soil extractions were performed by weighing out 0.5 g of sample into a 50-mL Falcon tube wrapped in aluminum foil to prevent interaction with light. Ten milliliters of a 0.2 M ammonium oxalate and oxalic acid solution adjusted to pH 3 was added to each sample tube. Tubes were shaken in the dark for 24 h on a reciprocal shaker and centrifuged at 3600 rpm for 15 min the next day. Supernatant was separated into a new sterile 50-mL Falcon tube and stored in a 4 °C cold room until analyzed for Si, Al, Fe, and Mn by ICP-OES (Table S4).

Total elemental analysis was performed by Activation Laboratories, Ltd. (Ontario, Canada). Samples were analyzed using a lithium metaborate/tetraborate fusion followed by dissolution in nitric acid and determination of major oxides by ICP-OES (Table S5).

### Statistical Analyses

Statistical analyses were performed to understand the changes in biogeochemical properties with depth. Analyses included multivariate approaches including principal component analysis (PCA), *k*-means cluster analysis, and linear mixed effects regression where data from both cores were included in the same analysis and core was used as a random effect.

Initial analyses indicated little variation in the aggregate stability and mineral and total elemental composition among core samples (Tables S2 and S3). The PCA and cluster analyses thus focused on the biogeochemical data, including pH and soil nutrient analyses, the average soil DNA biomass, and the selective dissolution data. The selective dissolution data included oxalate-extractable Mn and Fe normalized to total elemental Mn and Fe (Mno:Mntotal and Feo:Fetotal) to help identify potential effects of anaerobic mineral transformations. The data were clustered into similar groups using *k*-means. Exploratory analyses indicated that four clusters best separated the data into unique groups with a near even distribution of the number of samples in each cluster. Biogeochemical parameter variation among clusters was compared using one-way ANOVA and Tukey HSD post hoc means comparison (*α* = 0.05).

Several core samples, NC19 and NC20, exhibited very high outlier concentrations of extractable Ca and S likely associated with the presence of trace amounts of gypsum. These data were excluded from the PCA and cluster analysis. Prior to PCA and cluster analyses, all biogeochemical data were transformed to normal distributions using a Box-Cox transformation and standardized to similar value ranges using *z*-scores. Data that were reported as below detection limits were populated with values one-half that of the lower detection limit.

All statistical analyses were performed using JMP Pro version 16.2.0, 2020 (JMP Statistical Discovery LLC., Cary, North Carolina).

## Results

### Soil DNA Biomass

**Fig. 2:**
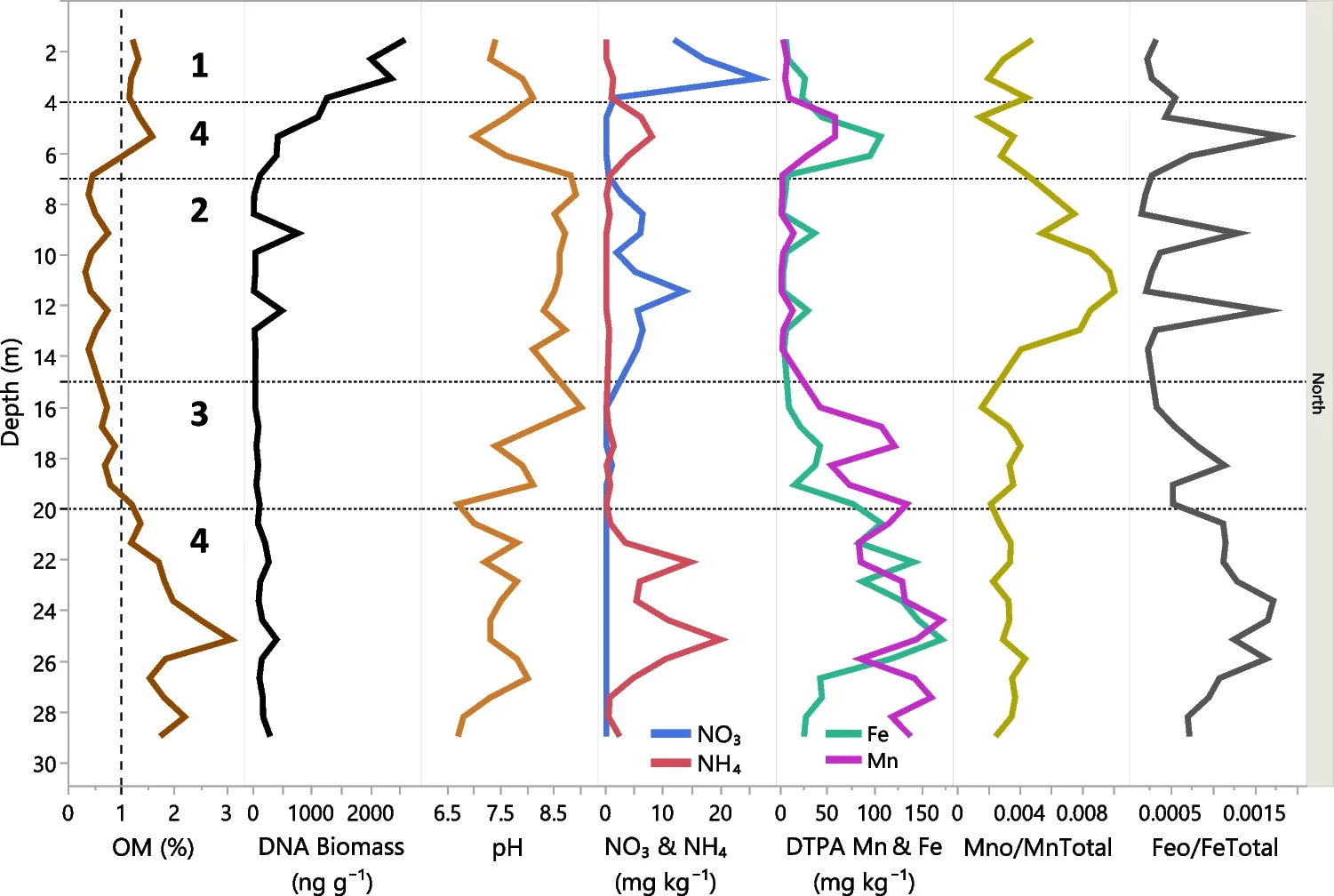
Depth profiles of biogeochemical properties for the north core including organic matter (OM), soil DNA biomass, pH 1:1 in water, KCl-extractable nitrate (NO_3_-N) and ammonium (NH4-N_-_), DTPA-extractable manganese (Mn) and iron (Fe), and the ratio of oxalate-extractable Mn to total elemental Mn (Mno:Mntotal) and oxalate-extractable Fe to total elemental Fe (Feo:Fetotal). The vertical dashed line in the organic matter panel marks 1%. The horizontal dashed lines denote breaks in clustered redox groups, labeled clusters 1, 2, 3, and 4

**Fig. 3:**
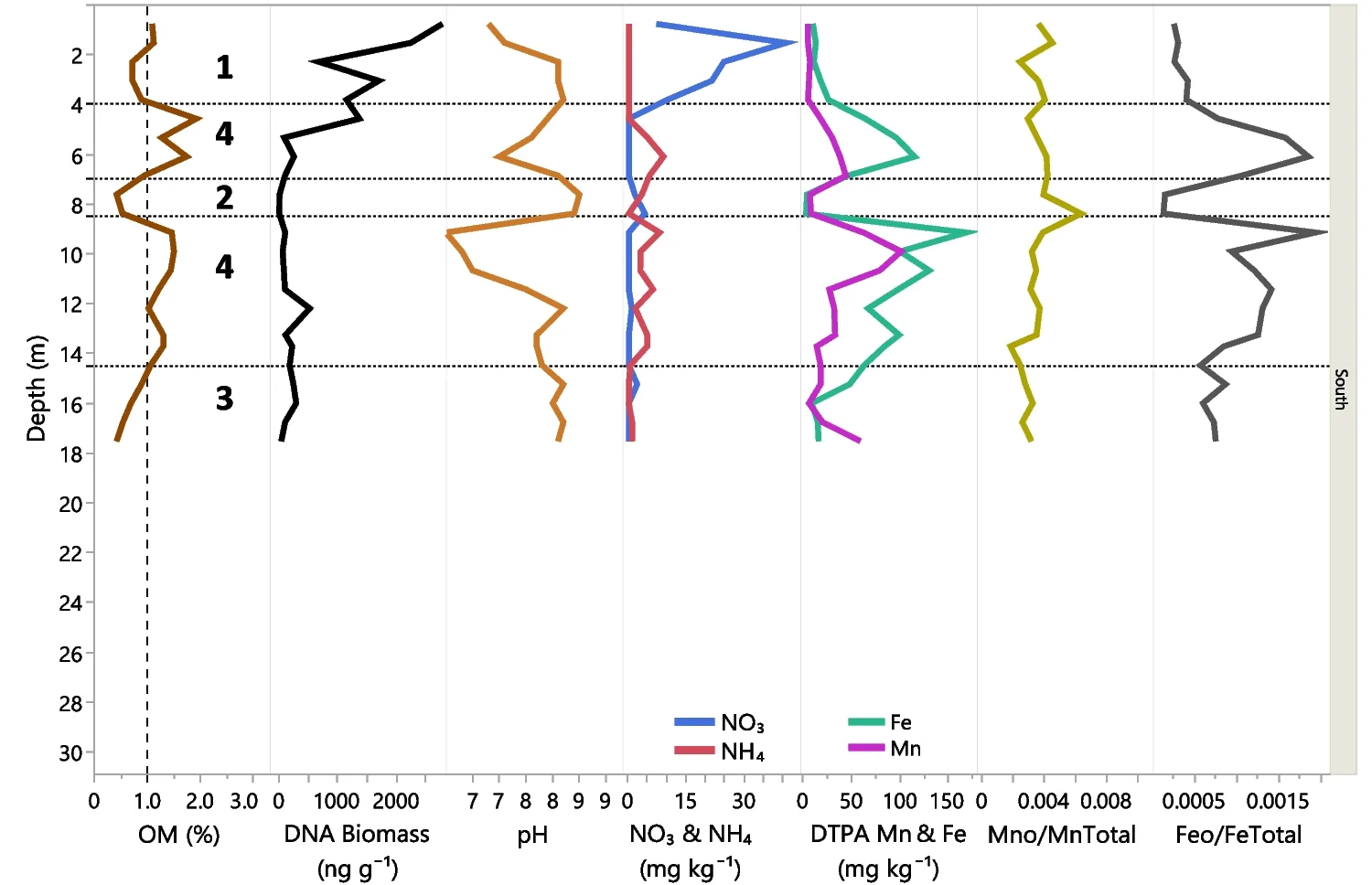
Depth profiles of biogeochemical properties for the south core including organic matter (OM), soil DNA biomass, pH 1:1 in water, KCl-extractable nitrate (NO_3_-N) and ammonium (NH_4_-N), DTPA-extractable manganese (Mn) and iron (Fe), and the ratio of oxalate-extractable Mn to total elemental Mn (Mno:Mntotal) and oxalate-extractable Fe to total elemental Fe (Feo:Fetotal). The vertical dashed line in the organic matter panel marks 1%. The horizontal dashed lines denote breaks in clustered redox groups, labeled clusters 1, 2, 3, and 4

Maximum soil DNA biomass was found in the upper 1.5 m in both NC and SC, with values of 2575 and 2781 ng/g, respectively (Figs. 2 and 3). Soil DNA biomass levels decreased substantially with increasing depth below the surface in both cores, with values < 1000 ng/g at depths greater than 5 m. The NC did exhibit two spikes in DNA biomass at 9 and 12 m, and the SC also exhibited a spike in DNA biomass at 12 m, increasing from values < 500 to nearly 1000 ng/g. Below ~ 13 m in depth, DNA biomass decreased below 500 ng/g and remained below this value through the remainder of both core depth profiles. The lowest measured soil DNA biomass were 3.5 and 4.7 ng/g in NC and SC, respectively, both at a depth of ~ 8 m.

### Biogeochemical Variability

Biogeochemical parameters varied with depth in both cores (Figs. 2 and 3). Specifically, %OM did not show a consistent decrease with depth but rather was highly variable across both cores. In the NC, values approached 3% OM at 25 m, and values were near 2% at 5 and 10 m in the SC. pH values ranged between 6 and 9 in both cores, with lower pH generally associated with zones of OM enrichment. Inorganic extractable nitrogen values were also highly variable with depth, with a general trend of greater NO_3_-N in surface layers down to ~ 4 m, after which values tended to decrease with depth and decreased below the detection limit of 0.5 mg/kg below 16 m in NC and 5 m in SC. Nitrate values were elevated between 10 and 14 m in the NC. Soil NH_4_-N concentrations ranged from 0.6 to 19.9 mg/kg in NC, with the highest concentration at 25 m. The NH_4_-N concentrations in SC ranged from 0.6 to 9.2 mg/kg with the highest concentration at 6 m. DTPA-Mn and DTPA-Fe also varied substantially with depth. Increased values of both were associated with increased values of %OM. Olsen P ranged from 0.5 to 15 mg/kg, and WEOC ranged from 53 to 380 mg/kg across both cores (Table S1). Both Olsen P and WEOC depth variation closely followed depth variation in %OM. Water-extractable nitrogen values ranged from 6.5 to 60 mg/kg and followed similar depth variation to NO_3_-N.

### Soil Texture and Water Stable Aggregates

Soil particle size distribution in both cores ranged from 25 to 43% silt + clay and a maximum sand percentage of 75% (Table S2). The last sample depth at 30 m in the NC reached into the native soil below the stockpile and had a much higher silt + clay of 58%. Water stable macroaggregate percentages varied from 0.1 to 3.5% in both cores, with a higher percentage of macroaggregates between 10 and 15 m in NC and 5 and 10 m in SC (Table S2).

### Mineral and Elemental Composition

**Fig. 4:**
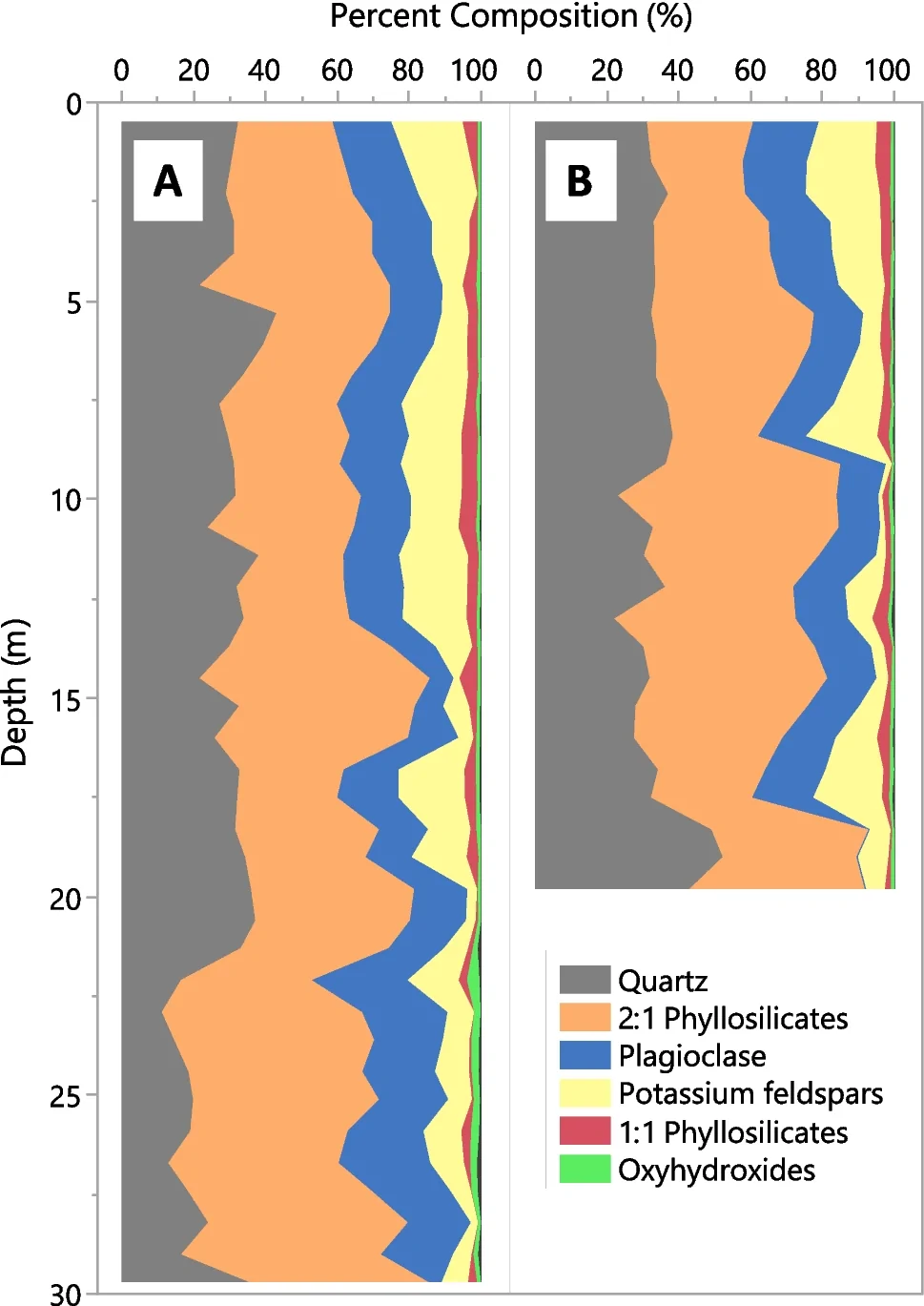
Mineral composition as determined by quantitative X-ray diffraction for the **A** north core and **B** south core

Mineral composition throughout both soil cores showed minimal variation with depth (Fig. 4). Quantitative X-ray analyses revealed the mineral composition was dominated by 2:1 phyllosilicates (primarily illite) and quartz in both cores. Total elemental analysis results were used to calculate several indices to quantify the degree of weathering of the stockpile material. The chemical index of weathering (CIW) calculated as $\mathrm{CIW}=\frac{{\mathrm{Al}}_{2}{\mathrm{O}}_{3}}{{\mathrm{Al}}_{2}{\mathrm{O}}_{3}+\text{ CaO}+ {\mathrm{Na}}_{2}\mathrm{O}} \times 100$ (Harnois, 1988) exhibited minimal variation with depth or among cores, indicating the parent material and degree of weathering were similar throughout both cores (Table S5).

Selective dissolution extracts indicated depth variation in ammonium oxalate–extractable Fe and Mn (Table S4). Oxalate-extractable metals were normalized relative to the total Fe and Mn from the total elemental analysis to isolate potential depth variation associated with biogeochemical changes (Figs. 2 and 3). Mno:Mntotal exhibited greater values at depths between 10 and 14 m in NC and a small relative spike near 8 m in SC. Values of Feo:Fetotal increased and decreased with values of %OM.

### Statistical Analyses

#### Principal Component Analyses

**Fig. 5:**
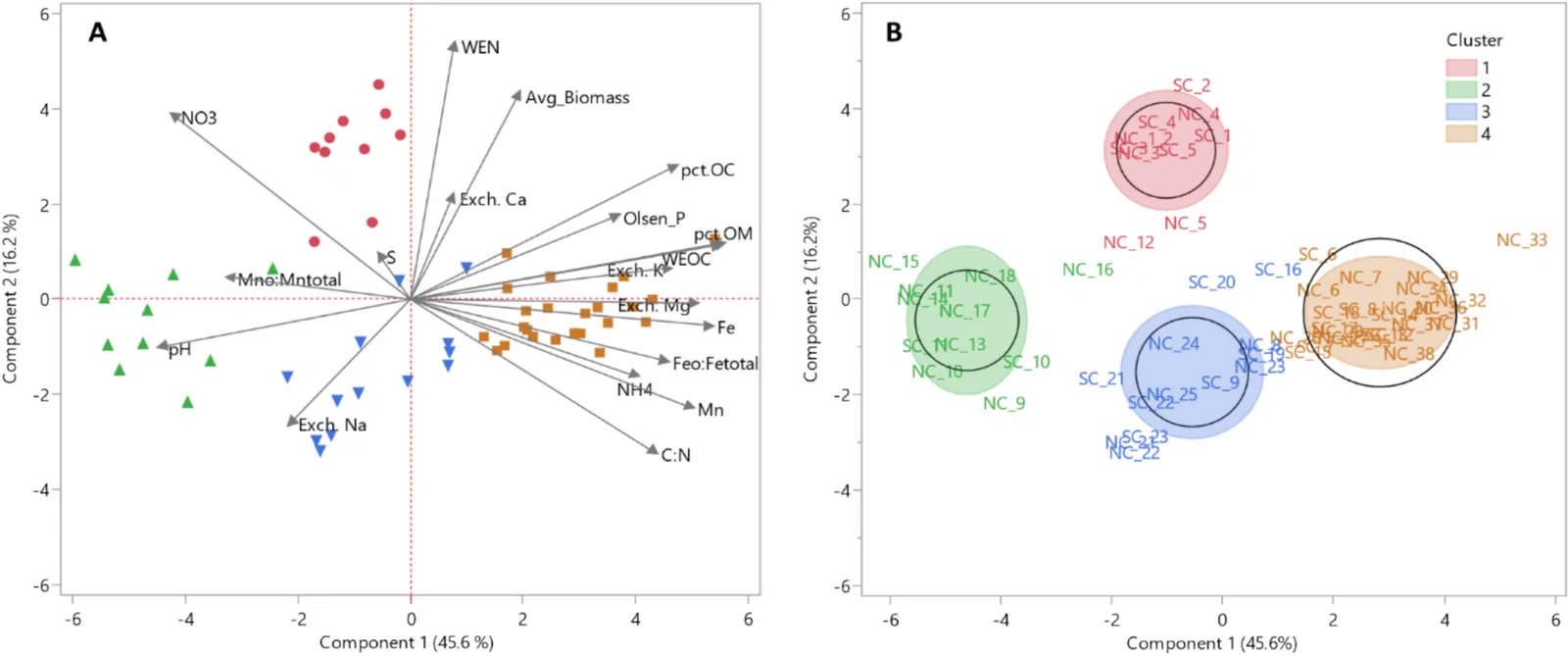
Principal component analyses on chemical and biological data with outliers omitted (**A**) and *k*-means cluster analysis with samples labeled by core (NC, north core; SC, south core) and depth (**B**). Circles drawn around the cluster centers are proportional to the number of samples in each cluster. The shaded area is the density contour around the mean indicating where 90% of the observations in that cluster would fall

The first two principal components accounted for over 61% of dataset variance, with 45.6% explained by the first principal component (Fig. 5A). The dominant factors contributing to the first principal component included %OM, WEOC, DTPA-Mn and Fe, pH, C:N, NO_3_-N and NH_4_-N, and Feo:Fetotal based on eigenvector coefficients. Component 2 was largely explained by WEN, DNA biomass, and NO_3_-N.

*K*-means cluster analyses indicated four clusters effectively separated the data into significantly different groups (Fig. 5B). Average values and means comparison across cluster groupings indicated variation in key biogeochemical properties (Table 1). The clusters were identified as clusters 1–4 and were characterized according to within-group property variation. Cluster 1 included samples with significantly greater DNA biomass, NO_3_-N and WEN, %OM near 1.0%, and lower amounts of DTPA-Mn and Feo:Fetotal. Cluster 2 was characterized as having very low DNA biomass, %OM, WEOC, and DTPA-Mn and DTPA-Fe, with relatively high pH and NO_3_-N. Cluster 3 exhibited relatively high values of WEOC and DTPA-Mn and DTPA-Fe, moderate %OM, and significantly lower WEN than the other groups. Cluster 4 had significantly greater %OM, WEOC, NH_4_-N, DTPA-Mn and DTPA-Fe, and Feo:Fetotal and significantly lower NO_3_-N and pH than the other groups. In addition to biogeochemical properties, depth was another key factor in sample clustering and segregation, particularly for cluster 1 that consisted only of samples from the upper 4 m of both cores. The range of depths for clusters 2–4 was highly variable and varied with OM content. Cluster 2 fell between 7 and 15 m below the core surface, Cluster 3 samples mainly originated from depths below 15 m, and cluster 4 samples occurred at depths as shallow as 5 m and as deep as 30 m.

**Table 1:** Mean values and standard deviations for key biogeochemical properties across clusters identified by k-means analysis*

	Cluster 1	Cluster 2	Cluster 3	Cluster 4
	*n*=10	*n*=11	*n*=13	*n*=24
Average Biomass (ng/g)	1747 ± 757 A	66 ± 141 C	164 ± 154 B	253 ± 318 B
OM (%)	1.0 ± 0.2 B	0.5 ± 0.1 C	0.8 ± 0.2 B	1.7 ± 0.4 A
WEOC (mg/kg)	130 ± 24.6 B	63.1 ± 8 C	109 ± 27.8 B	196.9 ± 60.3 A
WEN (mg/kg)	31.1 ± 14.3 A	13.6 ± 6 B	9.5 ± 2.1 C	14.1 ± 2.1 B
NO_3_ (mg/kg)	16.8 ± 11.8 A	5.0 ± 3.5 A	0.5 ± 0.6 B	0.3 ± 0 C
NH_4_ (mg/kg)	0.5 ± 0.4 B	0.7 ± 1.0 B	1.4 ± 1.6 B	5.9 ± 4.8 A
pH	7.8 ± 0.6 B	8.5 ± 0.2 A	8.1 ± 0.4 B	7.3 ± 0.5 C
Mn (mg/kg)	6.6 ± 3.1 C	3.9 ± 3.8 C	47.8 ± 34.9 B	89.8 ± 48.3 A
Mno/Mntotal (‰)	3.8 ± 1.1 B	7.0 ± 2.2 A	3.1 ± 0.7 B	3.1 ± 0.7 B
Fe (mg/kg)	17.7 ± 9.8 B	6.5 ± 7.6 C	37.2 ± 26.5 B	95.8 ± 40.9 A
Feo/Fetotal (‰)	0.43 ± 0.32 C	0.36 ± 0.43 C	0.77 ± 0.27 B	1.21 ± 0.43 A

*Values within a row labeled with different letters indicate significantly different values as determined by ANOVA and Tukey HSD (α=0.05). The ANOVA was performed using transformed and standardized variables. Note that untransformed means and standard deviations are reported in the table

## Discussion

Soil biogeochemical variation summarized by the cluster analysis indicated distinct zones of variable redox status that can be linked to the concentration of organic matter. Specifically, cluster 1 exhibited relative enrichment of organic matter, high DNA biomass, dominance of extractable nitrogen by nitrate, low levels of DTPA-extractable Fe and Mn, and moderately alkaline pH. The combination of these values indicates oxidized conditions with abundant microbial activity.

Cluster 2 samples generally exhibited organic matter below 1%, very low DNA biomass, alkaline pH, nitrate as the dominant extractable form of nitrogen, limited DTPA-Mn and DTPA-Fe, and significantly greater Mno:Mntotal than the other groups. These data suggest layers that remain oxidized despite their location at depths > 7 m. The oxidized state of these layers was likely a function of the lack of organic matter to serve as an energy source for heterotrophic anaerobes, thereby limiting microbial activity [40]. A lack of microbial activity was also suggested by the very low DNA biomass found in these layers.

Cluster 3 samples all occurred at depths below 4 m, contained moderate amounts of organic matter with values near 1%, contained relatively low DNA biomass, exhibited dominance of extractable nitrogen by ammonium, showed a decrease in pH relative to cluster 2, and contained significantly greater amounts of DTPA-Mn and DTPA-Fe and low amounts of Mno:Mntotal. This combined set of properties suggests layers that have undergone some degree of anaerobic alteration, enough to transform extractable nitrogen to ammonium and transform a significant fraction of the short-range-order Mn oxides to DTPA-extractable forms and shift the pH towards neutrality. Further reduction and anaerobic transformation were likely limited by organic matter abundance.

Finally, cluster 4 included layers below 4 m that were enriched in organic matter, were dominated by ammonium as the extractable form of nitrogen, exhibited near neutral pH values, and contained significantly greater DTPA-Mn and DTPA-Fe, significantly greater Feo:Fetotal, and relatively low values of Mno:Mntotal. These layers also coincided with samples that contained substantial amounts of decomposed particulate plant material and exhibited a strong unpleasant odor when extracted and subsampled in the field, likely associated with the presence of sulfide gases. These data all indicate strongly reducing conditions.

Stockpile biogeochemical alteration thus appeared to be associated with zones of variable redox state that corresponded with depth and organic matter content. Reduced conditions were evident at depths greater than 4 m where organic matter contents approached and were greater than 1%; however, oxidized conditions were evident all the way down to ~ 15 m when organic matter content was < 1%. These patterns indicate that organic matter was the dominant control on formation of anaerobic conditions. In suboxic and anoxic conditions where oxygen is limited, microbial metabolism is facilitated by alternative terminal electron acceptors, including, in order of decreasing energy yield, nitrogen, Mn, and Fe [41]. Reduction of nitrogen leads to denitrification and the transformation of nitrate to ammonium via dissimilatory nitrate reduction [42]. The alternate dominance of depth zones by nitrate or ammonium strongly suggests dissimilatory nitrate reduction occurred during stockpile storage (Figs. 2 and 3). However, ammonification via organic matter decomposition may have also contributed to increased NH_4_-N [43]. Williamson and Johnson [6] also observed significant increases in NH_4_-N concentrations in a 4-year-old topsoil stockpile at depths greater than 1 m that were driven by anaerobic conditions.

The increase in DTPA-Mn and DTPA-Fe was likely associated with solubilization of Mn and Fe oxides and partitioning to more labile, plant-available pools under anaerobic conditions [44, 45]. DTPA-Mn values exhibited a near 12-fold increase when comparing clusters 1 and 4, whereas DTPA-Fe values increased approximately fivefold (Table 1). Manganese undergoes reduction at redox potential values greater than those of Fe, so it was not unexpected to find greater change and sensitivity in the partitioning of Mn [46]. The concurrent reduction in Mno:Mntotal values in these layers suggests dissolution of short-range-order Mn oxides [47]. Plant-available Mn concentrations also vary significantly with pH, with increasing availability at lower pH values [40]. We observed a significant decrease in pH from alkaline values to near neutral in the reduced layers that would also increase plant-available Mn concentrations (Table 1). It is difficult to disentangle the dominant control in this system as reduction and pH change occur simultaneously, and indeed, a mixed-effects linear regression model where core (north or south) was included as random effect indicated that variation in DTPA-Mn was best explained by a combination of organic matter, pH, and depth (Table S6). DTPA-Mn also tended to increase with decreasing Mno:Mntotal, further suggesting redox-driven transformation.

Iron speciation behaved somewhat differently. While we observed an increase in labile, plant-available forms of Fe in the reduced layers, these layers also coincided with significantly greater values of Feo:Fetotal (Table 1). Cyclical redox fluctuations can lead to the dissolution and reprecipitation of short-range-order Fe-oxyhydroxides, with formation of short-range-order phases favored with greater organic matter [48]. Linear mixed-effects regression models indicated that the best predictor of both DTPA-Fe and Feo:Fetotal was organic matter content, with no significant correlation with pH (Table S6). Furthermore, unlike the noted inverse relationship noted for Mn partitioning, DTPA-Fe and Feo:Fetotal were positively correlated throughout both cores. It is thus less clear if the observed increased concentrations of labile Fe were the result of redox processes or simply associated with greater concentrations of organic matter in these layers.

Given these patterns and the core pH values ranging from 7 to 9, the data suggest redox potentials on the order of ~ 100–300 mV [49] in clusters 3 and 4. This is low enough to fully reduce N and Mn and potentially some forms of Fe [50]. The strong odor associated with cluster 4 samples during sampling suggests possibly even lower redox potentials and reduction of sulfate to sulfur gases [51]. Special attention should be paid to soil chemical alterations that result in higher extractable metal concentrations in topsoil stockpiles, especially under anoxic conditions where sulfidic gases and higher concentrations of Fe may become toxic for plant growth. High concentrations of soluble ferrous Fe in soil solution can create Fe toxicity for plants [52]. Abdul-Kareem and McRae [3] found elevated levels of methane, ethane, and ethylene alongside metal accumulation in topsoil stockpiles which they cite as problematic for plant growth. Future research should assess the effects of elevated soil gas level and soluble metal concentrations on seed germination and plant establishment when utilizing such substrates as capping materials for revegetation.

The role of redox status with depth may be integral to the measured DNA biomass as well. While surface stockpile layers had DNA biomass > 2500 ng/g, the concentrations dropped to less than 1000 ng/g below 4 m in both cores. Comparatively, undisturbed native topsoil near the sample site location averaged 6822 ± 2628 ng/g and up to 9250 ± 2123 ng/g in the soil root zone [32]. In contrast, undisturbed soils on two mine sites at lower elevations in the Arizona Sonoran Desert presented average biomass values of 2169 ± 2095 ng/g and 1933 ± 456 ng/g, respectively, indicating a range of ecosystem-sustaining biomass levels in arid ecosystems (personal communication, Julie Neilson). These patterns were similar to those observed in a 7.5-m-deep 25-year-old topsoil stockpile associated with surface coal mining in the Northern Great Plains of the US [8]. These authors found that surface layers of the stockpile exhibited microbial abundance nearly one-third less than the adjacent native topsoil, as well as a significant decrease in microbial abundance with increasing depth. The loss of large and diverse microbial populations with stockpiling may negatively affect biogeochemical cycling when these materials are used as capping substrate, limiting plant establishment and successful revegetation.

Although a decrease in microbial DNA biomass was observed alongside anaerobic conditions with increasing depth and organic matter contents as found in previous studies, the depth at which these transformations occur is far greater than previously recorded (here observed at depths of 6–22 m). Within the top 5 m of the stockpile, soil DNA biomass was preserved at reasonable levels to support vegetation despite 14 years of stockpiling [32]. Other studies have typically observed these effects starting at 0.5–1 m in depth [3, 8]. Some studies recommend stockpile height should be limited to 1 m or less to reduce anaerobic effects and compaction [53]. However, our study suggests that in semiarid or arid climatic conditions, stockpiles may potentially be constructed up to 15 m in depth before anaerobic transformation begins to occur if organic matter content is below 1%. This may be more practical in stockpile construction in this context given the scale of topsoil material cleared during open pit mining operations. Additionally, because chemical and biological transformations were largely coupled with organic matter contents, it is worth planning the construction of stockpiles based on the percentage of organic matter. A key recommendation to industry would be to not include grubbed vegetation in topsoil stockpiles as it was apparent from this study that the zones most enriched in organic materials and decayed plant parts coincided with zones of substantial biogeochemical change driven by anaerobic conditions. Plant materials should be harvested and stored separately or chipped for mulch or compost production.

The stockpiled topsoil will ultimately be used as capping materials for mine-land reclamation and to promote vegetation establishment. It is unknown how the stockpile materials will respond once they are re-mined, mixed, and deployed as capping materials. Specifically, it is unclear what biogeochemical changes will occur with the reintroduction of the organic matter–rich anoxic zone materials to an oxidizing surface environment or how mixing of anoxic and oxic layers may mask and/or dilute the potential detrimental effects of the anoxic material on plant establishment. Fully understanding these interactions requires further experimentation and plant establishment and germination studies under field conditions that were beyond the scope of the current study. However, in lieu of these experiments, we can approximate the effects of mixing on key biogeochemical properties (e.g., pH, available P, organic C, total N, NO_3_-N and NH_4_-N, and DNA biomass) by looking at average property values across the entire core depth and comparing these values with local undisturbed soils to understand how they may differ from the native environment (Table S1). Compared to undisturbed soil data from the mine site [32], the stockpile materials exhibited greatly reduced values of biogeochemical properties important for plant growth including soil microbial DNA biomass (average of 438 ± 687 ng/g relative to 9250 ± 2123 ng/g), available P (4.5 ± 3.5 mg/kg relative to 22.5 ± 5.5 mg/kg), organic C (0.4 ± 0.3% relative to 1.4 ± 0.3%), total N (0.03 ± 0.01% relative to 0.13 ± 0.04%), and NO_3_-N (3.8 ± 7.5 mg/kg relative to 6.7 ± 2.5 mg/kg). The only soil nutrient concentration greater on average in the stockpile material was NH_4_-N that averaged 2.8 ± 3.9 mg/kg relative to 0.9 ± 0.9 mg/kg in the undisturbed native soil, although it is likely that much of the NH_4_-N will oxidize to NO_3_ when mixed and exposed to aerobic conditions at the soil surface. Even though the average stockpile values are generally lower than that of adjacent native soil, chronosequence studies of plant establishment on degraded soils in mine-lands have indicated that initial plant establishment enhances soil microbial abundance and diversity and builds soil nutrient stores that over time can approach levels measured in native soils [11, 12, 28, 32].

## Conclusion

This study presents some of the first data documenting how decadal scale stockpiling of topsoil up to depths of 30 m affects soil biogeochemical properties in the semiarid environment of the Southwest US. The results from this study demonstrated that significant alteration of soil biogeochemical, mineral, and microbial properties occurred during 14 years of topsoil stockpile storage. The 30-m-deep stockpile was characterized into four zones of varying redox status, with highly reducing zones associated with layers where organic matter content was greater than 1%. The highly reduced zones occurred at depths as shallow as 4 m and as deep as 20 m. However, we also observed evidence that layers even as deep as 15 m remained oxidized, despite the deep burial and relatively long storage time, as long as the organic matter percent was below 1%. Our findings suggest that topsoil stockpiles may be constructed to relatively deep depths in this semiarid environment if organic matter content is kept below a concentration threshold of ~ 1%. Vegetation harvested prior to and during stockpile construction should be managed and stored separately and not directly incorporated into mineral topsoil stockpile. This will limit the potential for development of anaerobic conditions that can lead to deleterious shifts in soil health that degrade its potential for supporting reclamation and revegetation efforts. Future and ongoing research will examine how well the stockpiled topsoil material supports plant growth and vegetation establishment when mixed and deployed as capping materials.

## Supplementary Information

Below is the link to the electronic supplementary material.

Supplementary file1 (PDF 492 KB)

Supplementary file2 The supplemental materials include tables of all collected data. (XLSX 52 KB)
