# Supplementary material for: Effects of Stockpiling on Topsoil Biogeochemistry for Semiarid Mine Reclamation
Source: Min Metall Explor. 2025 Jan 8;42(1):15–26. doi: 10.1007/s42461-024-01164-2 (PMC11787246; doi:10.1007/s42461-024-01164-2)
Supplement: Supplementary file 1 — Supplementary file1 (PDF 492 KB) [file 42461_2024_1164_MOESM1_ESM.pdf]

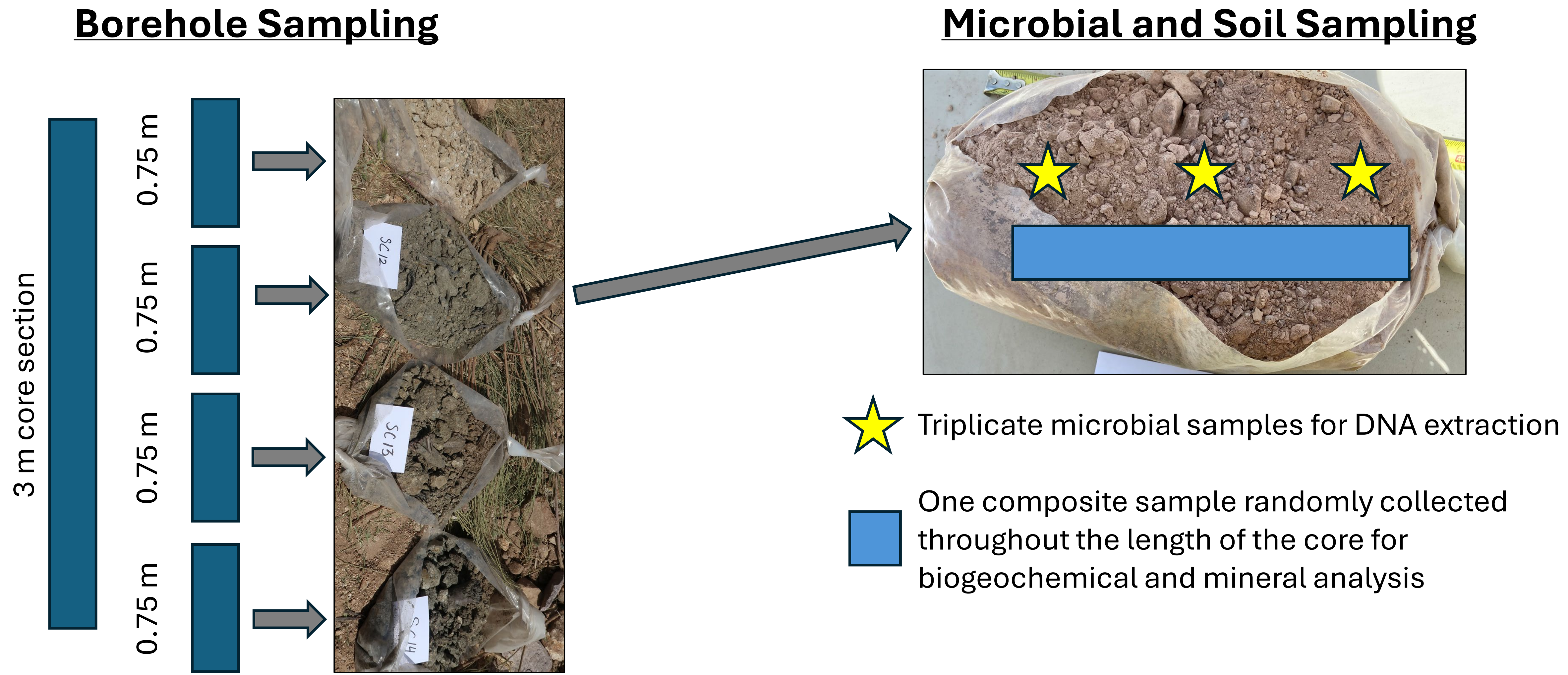

Bore holes were drilled through the entire thickness of the stockpile. Core sections were collected in 3-m sections and each section extruded into 0.75 m subsections. The 0.75 subsections represent the sample depths for microbial and biogeochemical analysis. Microbial samples were collected in triplicate from each sub-core. These samples were collected 10 cm from the top of the sub-core, in the center of the sub-core, and 10 cm from the bottom of the sub-core using sterilized scoopulas. The triplicate samples were averaged to provide one value of DNA biomass per subsection. Soil samples for biogeochemical and mineralogical analyses were randomly collected from the entire length of the subsection into one composite sample per subsection. These samples were sieved through a 4.75 mm sieve to separate large rocks from soil material and the rock weight recorded in the field. Sieved samples were homogenized and collected into two Whirlpack packs and saved for biogeochemical and mineralogical analyses.
